# TTPAL promotes gastric tumorigenesis by directly targeting NNMT to activate PI3K/AKT signaling

**DOI:** 10.1038/s41388-021-01838-x

**Published:** 2021-10-12

**Authors:** Wenxiu Liu, Hongyan Gou, Xiaohong Wang, Xiaoming Li, Xiaoxu Hu, Hao Su, Shengmian Li, Jun Yu

**Affiliations:** 1 Shenzhen Research Institute, The Chinese University of Hong Kong, Shenzhen, China; 2 Department of Gastroenterology and Hepatology, The Fourth Hospital of Hebei Medical University, Shijiazhuang, China; 3 Institute of Digestive Disease and Department of Medicine and Therapeutics, State Key laboratory of Digestive Disease, Li Ka Shing Institute of Health Sciences, The Chinese University of Hong Kong, Hong Kong, China

**Keywords:** Gastric cancer, Prognostic markers

## Abstract

Copy number alterations are crucial for gastric cancer (GC) development. In this study, Tocopherol alpha transfer protein-like (TTPAL) was identified to be highly amplified in our primary GC cohort (30/86). Multivariate analysis showed that high TTPAL expression was correlated with the poor prognosis of GC patients. Ectopic expression of TTPAL promoted GC cell proliferation, migration, and invasion in vitro and promoted murine xenograft tumor growth and lung metastasis in vivo. Conversely, silencing of TTPAL exerted significantly opposite effects in vitro. Moreover, RNA-sequencing and co-immunoprecipitation (Co-IP) followed by liquid chromatograph-mass spectrometry (LC-MS) identified that TTPAL exerted oncogenic functions via the interaction of Nicotinamide-N-methyl transferase (NNMT) and activated PI3K/AKT signaling pathway. Collectively, TTPAL plays a pivotal oncogenic role in gastric carcinogenesis through promoting PI3K/AKT pathway via cooperating with NNMT. TTPAL may serve as a prognostic biomarker of patients with GC.

## Introduction

Gastric cancer (GC) represents an important global health problem. GC is the fifth commonest cancer worldwide and the third leading cause of cancer-related death [1]. GC is a complex and highly heterogeneous disease, largely because it arises from multiple interaction of genetic alterations, epigenetic changes, infection, and the tumor microenvironment. Pathways that contribute to gastric carcinogenesis and underline driver genes have been identified based on the molecular characteristics and are potential therapeutic targets [2]. The identification of the novel oncogene and its related signaling pathway will help to recognize novel therapeutic target. Copy number alterations are common somatic changes in cancer featured with gain or loss in copies of DNA sections [3]. Recurrent gain and amplification of the long arm of chromosome 20 (20q) has been observed in 70% of primary GC [4]. Using whole genome sequencing we identified that tocopherol alpha transfer protein-like (TTPAL), located at 20q13.12, was amplified in colorectal cancer. TTPAL promoted colorectal tumorigenesis by activating Wnt/β-catenin signaling [5]. By analysis the public The Cancer Genome Atlas (TCGA) database, we found that TTPAL was frequently amplified in GC and positively correlated with its upregulated copy number variation. However, whether the contribution of TTPAL in the progression of GC is still unclear. In this study, we revealed that TTPAL exerted oncogenic role in GC, elucidated the regulatory context of TTPAL by activating PI3K/AKT signaling pathway. TTPAL high expression was associated with poorer survival of GC patients. Moreover, TTPAL was a potential therapeutic target in GC.

## Results

### TTPAL was frequently amplified in GC primary tissues and associated with poor survival of GC patients

**Fig. 1:**
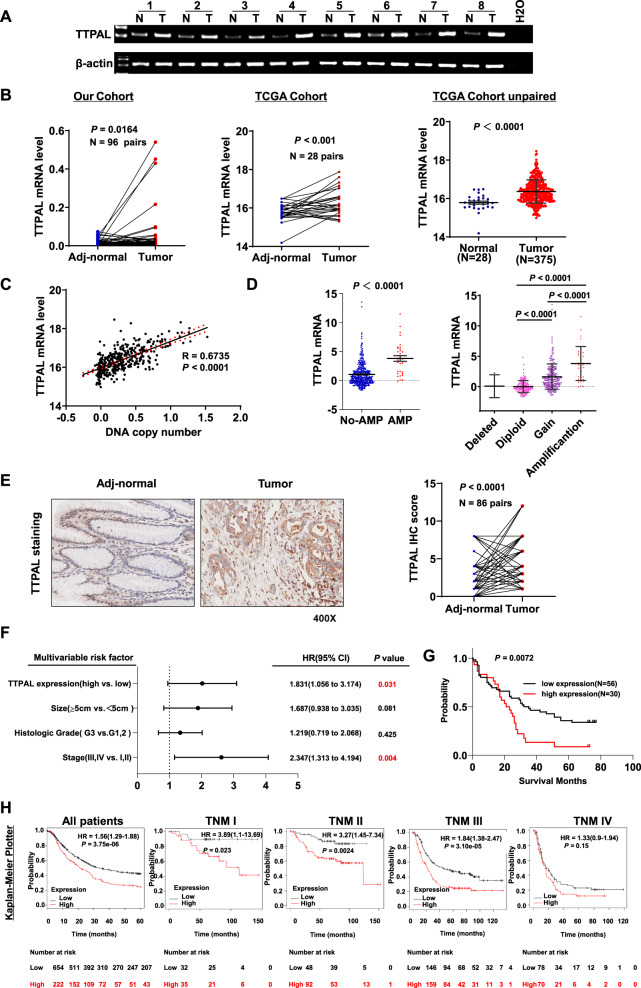
TTPAL was overexpressed in GC tissues and associated with poor survival of patients. **A** TTPAL mRNA expression was upregulated in GC compared to paired adjacent normal tissues as shown by RT-PCR (Our cohort I, from Beijing). **B** TTPAL mRNA expression was upregulated in GC compared to paired adjacent normal tissues as shown by qRT-PCR (Our cohorts II, from Shijiazhuang) and RNA-seq data from TCGA study also showed upregulation of TTPAL in GC as compared to adjacent normal tissues (paired and unpaired samples). **C** TTPAL copy number was positively correlated with its mRNA expression in TCGA cohort by the Pearson correlation coefficient analysis. **D** TTPAL mRNA expression was higher in TTPAL DNA copy number amplification group compared with no amplification group. **E** TTPAL protein expression was significantly higher in primary GC as compared to adjacent normal tissues as shown by IHC staining (Our cohort Ш, from Shanghai). **F** Multivariate Cox regression analysis showed that TTPAL expression was an independent poor prognostic factor for GC patients (relative risk (RR) = 1.831, 95% CI: 1.056–3.174, *p* = 0.031). **G** Kaplan–Meier survival analysis showed GC patients with high TTPAL expression had poorer survival than those with low TTPAL expression at protein level from Our cohort Ш. **H** Prognostic value of TTPAL expression was validated in GC patients from Kaplan–Meier plotter (http://kmplot.com/).

TTPAL mRNA expression was significantly upregulated in GC as compared with their adjacent non-tumor tissues in our cohort (*N* = 96, *p* = 0.0164) and was confirmed in paired GC tumor tissues as compared to adjacent normal controls (*N* = 28) and in TCGA cohort in GC tumor tissues (*N* = 375) compared with normal controls (*N* = 28) (Fig. 1A, B). TTPAL mRNA expression was positively correlated with DNA copy number (*R* = 0.6735, *p* < 0.0001) (Fig. 1C). TTPAL mRNA expression was higher in TTPAL DNA copy number amplification group compared with no amplification group (*N* = 412, *p* < 0.0001) (Fig. 1D). In keeping with mRNA expression, TTPAL protein expression was also significantly higher in primary gastric tumors as compared to adjacent non-tumor tissues by immunohistochemical (IHC) staining (*N* = 86, *p* < 0.0001) (Fig. 1E).

We further evaluated the clinicopathological and prognostic significance of TTPAL expression in patients with GC. High TTPAL expression predicted a higher risk of cancer-related death by univariate Cox regression analysis (relative risk (RR) = 1.832, 95% CI: 1.075–3.122, *p* = 0.026) (Supplementary Table 1). Multivariate Cox regression analysis showed that TTPAL expression was an independent poor prognostic factor for GC patients (RR = 1.831, 95% CI: 1.056–3.174, *p* = 0.031) (Fig. 1F). Kaplan–Meier survival curves showed that GC patients with high TTPAL expression had significantly shorter survival than those with low TTPAL expression (*p* = 0.0072) (Fig. 1G). This finding was further validated in GC samples from Kaplan–Meier plotter (*p* < 0.0001) (Fig. 1H). Moreover, after stratified by tumor staging, TTPAL overexpression predicted poor prognosis in stages I–III GC patients, but not for stage IV GC patients (Fig. 1H). No correlation was found between TTPAL expression and other clinicopathological features such as age, gender, tumor differentiation, and tumor nodes metastasis (TNM) stage (Supplementary Table 2).

### Ectopic expression of TTPAL promoted cell growth and cell cycle progression

**Fig. 2:**
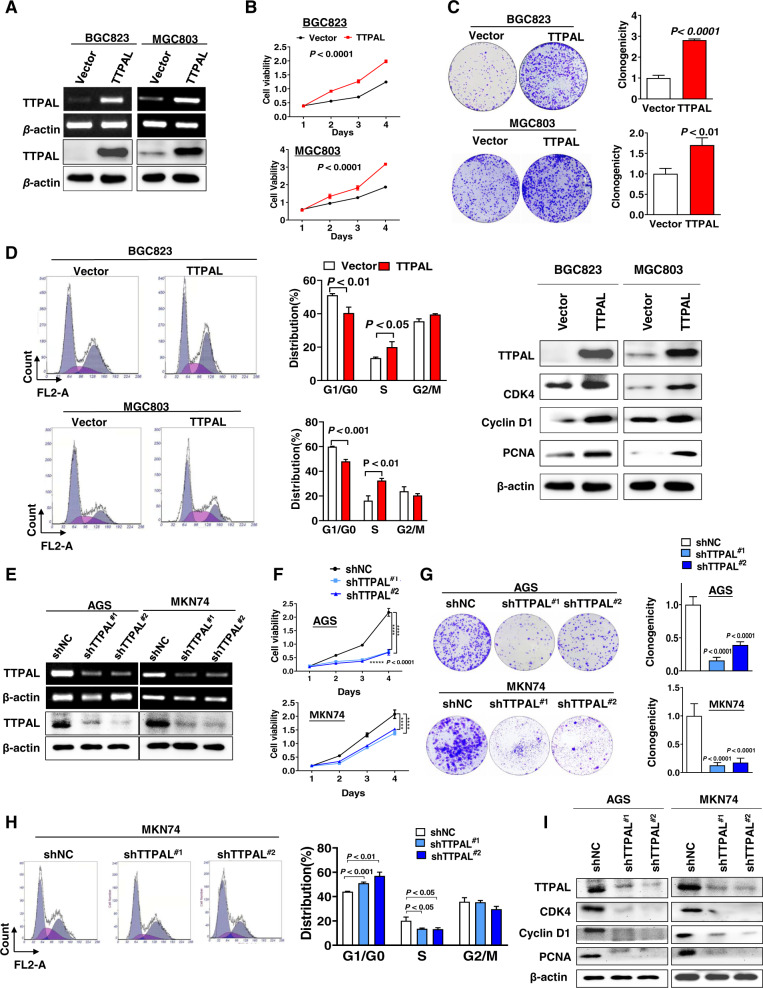
TTPAL promoted cell growth and cell cycle progression. **A** Ectopic expression of TTPAL in BGC823 and MGC803 cells, confirmed by RT-PCR and western blot. **B** TTPAL expression significantly increased cell viability. **C** TTPAL expression significantly increased cell clonogenicity. **D** TTPAL expression significantly promoted cell cycle G1-S transition and enhanced protein levels of cyclin D1, CDK4, and PCNA. **E** Knockdown of TTPAL by shTTPAL in AGS and MKN74 cells was confirmed by RT-PCR and Western blot. **F** TTPAL knockdown significantly inhibited cell viability. **G** TTPAL knockdown significantly inhibited cell clonogenicity. **H** TTPAL knockdown caused G1-S arrest. **I** Knockdown of TTPAL decreased protein levels of cyclin D1, CDK4, and PCNA.

To investigate the function of TTPAL in GC, TTPAL expression vector or empty vector was stably transfected into cells with GC cells (BGC823 and MGC803) with low TTPAL expression (Supplementary Fig. 1). Ectopic expression of TTPAL was confirmed by RT-PCR and western blot (Fig. 2A). Ectopic expression of TTPAL significantly promoted cell viability (*p* < 0.0001) (Fig. 2B) and clonogenicity in BGC823 (*p* < 0.0001) and MGC803 (*p* < 0.01) (Fig. 2C). We next investigated the effect of TTPAL on cell cycle by flow cytometry. Ectopic expression of TTPAL accelerated G1/S phase progression in BGC823 (*p* < 0.05) and MGC803 cells (*p* < 0.01) (Fig. 2D). The enhanced G1/S cell cycle by TTPAL was confirmed by the upregulation of the key G0/G1 phage regulators CDK4, cyclin D1 and S phase marker PCNA (Fig. 2D). Conversely, knockdown of TTPAL by RNA interference in AGS and MKN74 cells (Fig. 2E) markedly inhibited cell viability (*p* < 0.0001) (Fig. 2F) and clonogenicity (*p* < 0.0001) (Fig. 2G), decelerated the G1-S cell cycle transition (*p* < 0.05) (Fig. 2H) and downregulated the protein levels of CDK4, cyclin D1, and PCNA (Fig. 2I). Therefore, TTPAL promoted cell proliferation in GC through promoting G1-S cell cycle progression.

### TTPAL promoted migration and invasion of GC cells

**Fig. 3:**
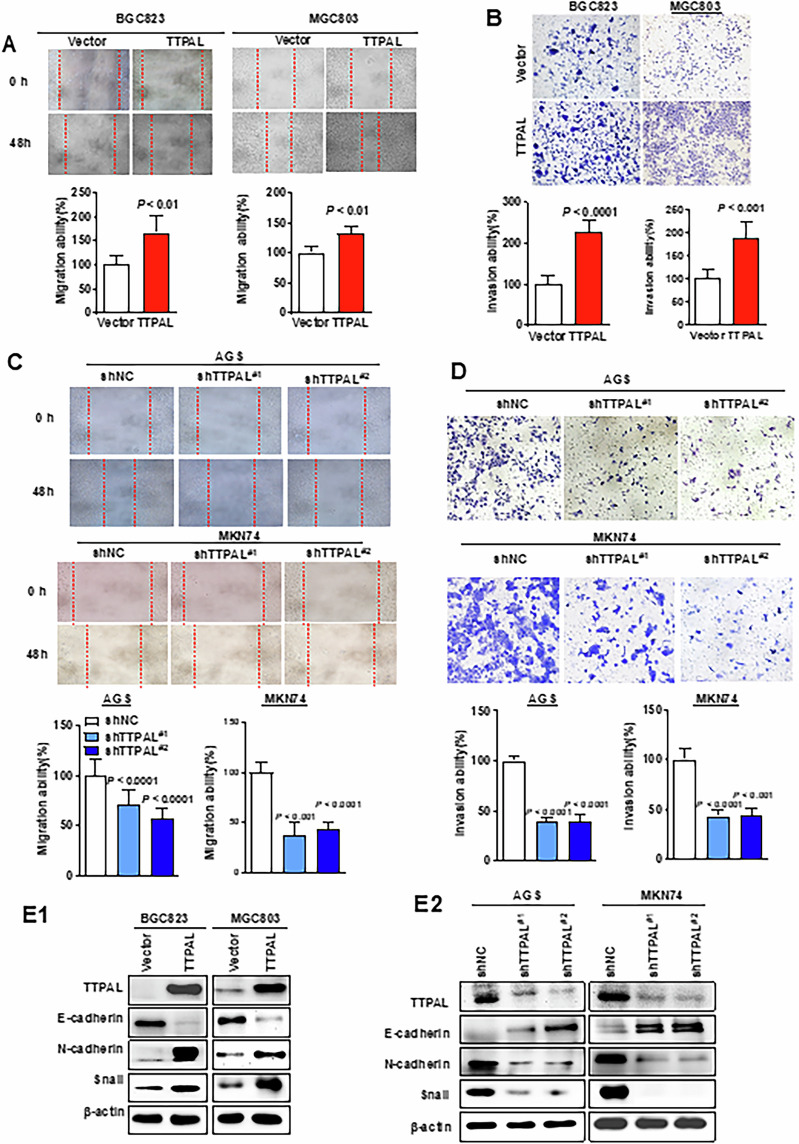
TTPAL promoted migration and invasion of GC cells. **A** Representative images of wound healing assay revealed that ectopic expression of TTPAL promoted cell migration in BGC823 and MGC803 cells. **B** Representative images of matrigel invasion transwell assay revealed that ectopic expression of TTPAL promoted cell invasion in BGC823 and MGC803 cells. **C** Knockdown of TTPAL significantly inhibited migration ability. **D** Knockdown of TTPAL significantly decreased cell invasion ability. **E** TTPAL expression increased the levels of N-cadherin and Snail, and decreased E-cadherin level in BGC823 and MGC803 cells, while knockdown of TTPAL showed the opposite effect on these EMT markers.

Ectopic expression of TTPAL promoted the cell migration and invasion by wound healing assay (*p* < 0.01) (Fig. 3A) and matrigel invasion assays in BGC823 (*p* < 0.0001) and MGC803 (*p* < 0.001) cells (Fig. 3B), respectively. Conversely, TTPAL knockdown in AGS and MKN74 cells exerted opposite effects on cell migration (*p* < 0.0001) (Fig. 3C) and invasion (*p* < 0.0001) (Fig. 3D). TTPAL promoted the epithelial–mesenchymal transition (EMT) through upregulation of mesenchymal markers (N-cadherin and Snail) and downregulation of epithelial markers (E-cadherin), as shown by western blot (Fig. 3E1). In contrast, knockdown of TTPAL showed the opposite effect on these EMT markers (Fig. 3E2). These findings demonstrated that TTPAL also functioned in regulating cell migration and invasion of GC cells.

### TTPAL activated PI3K/AKT signaling

**Fig. 4:**
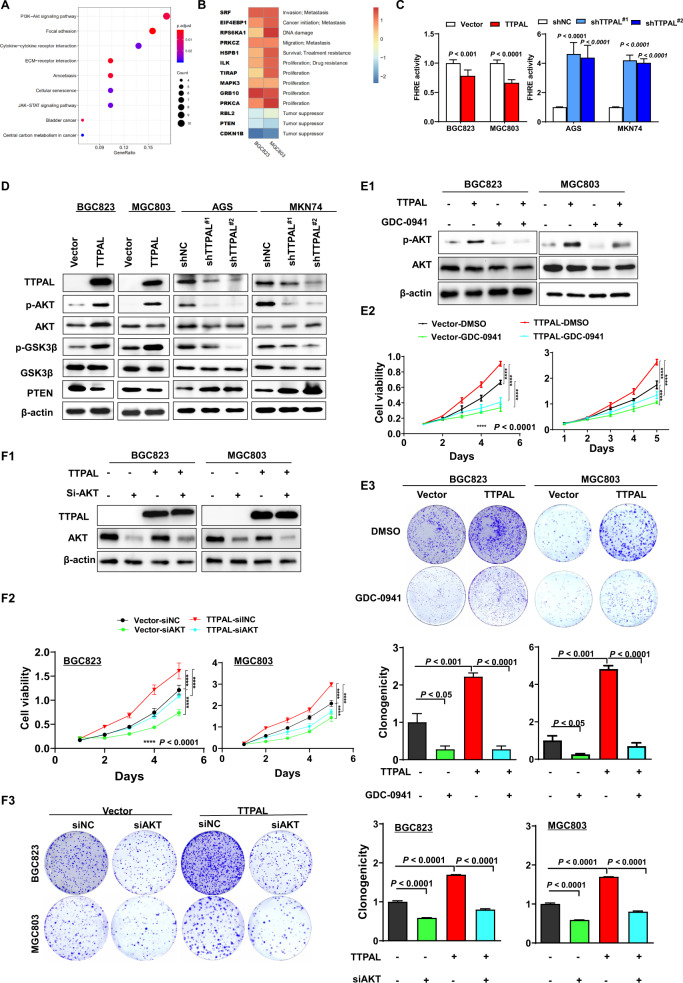
TTPAL activated PI3K/AKT signaling. **A** KEGG pathways enriched by differentially expressed genes affected by TTPAL. **B** Downstream targets of TTPAL identified by PI3K/AKT signaling pathway PCR array. **C** Ectopic expression of TTPAL significantly reduced FOXO reporter activity while knockdown of TTPAL significantly suppressed PI3K/AKT activity. **D** Western blot results showed that TTPAL expression enhanced the expression of PI3K/AKT signaling-related markers, while knockdown of TTPAL showed the opposite effect. **E** PI3K/AKT signaling inhibitor GDC-0941 treatment abolished the promoting effects of TTPAL on cell proliferation and clonogenicity (BGC823, 0.5 μmol/l and MGC803, 1 μmol/l). **F**. AKT knockdown also abolished the tumor-promoting function of TTPAL.

To understand the molecular basis of the oncogenic property of TTPAL, we performed RNA-sequencing in TTPAL overexpression and control GC cells. KEGG pathway enrichment analysis showed that TTPAL significantly activated PI3K/AKT signaling (Fig. 4A). We further confirmed the result by PCR array analysis. The PCR array result showed, ectopic expression of TTPAL activated the expression of PI3K/AKT signaling-related genes while downregulated tumor suppressors, such as RBL2, PTEN, and CDKN1B (p27) [6, 7] (Fig. 4B). Consistently, TTPAL expression positively correlated with multiple target genes of PI3K/AKT signaling in primary GC tissues from TCGA cohort, including SRF, EIF4EBP1, RPS6KA1, PRKCZ, ILK, TIRAP, MAPK3, GRB10, and PRKCA. On the other hand, TTPAL expression was negatively correlated with PTEN (Supplementary Fig. 2).

Ectopic expression of TTPAL significantly activated PI3K/AKT signaling as evidenced by reducing FOXO reporter activity. Conversely, TTPAL knockdown suppressed PI3K/AKT activity (Fig. 4C). Consistently, Ectopic expression of TTPAL increased phospho-AKT(p-AKT), phospho-glycogen synthase kinase-3beta and decreased the protein level of PTEN while knockdown of TTPAL showed the opposite effects (Fig. 4D).

To test whether the oncogenic function of TTPAL was depended on PI3K/AKT activation, GC cells with or without ectopic expression of TTPAL were treated with PI3K/AKT inhibitors GDC-0941 [8]. GDC-0941 treatment inhibited AKT phosphorylation (Fig. 4E1) and abolished growth promoting effect induced by TTPAL expression, as evidenced by cell viability (Fig. 4E2) and colony formation assays (Fig. 4E3). Meanwhile, silence of AKT (Fig. 4E1) can also abolished growth promoting effect of TTPAL on cell viability (Fig. 4E2) and colony formation (Fig. 4E3), indicating that TTPAL promotes GC by activating PI3K/AKT pathway.

### NNMT directly interacted with TTPAL

**Fig. 5:**
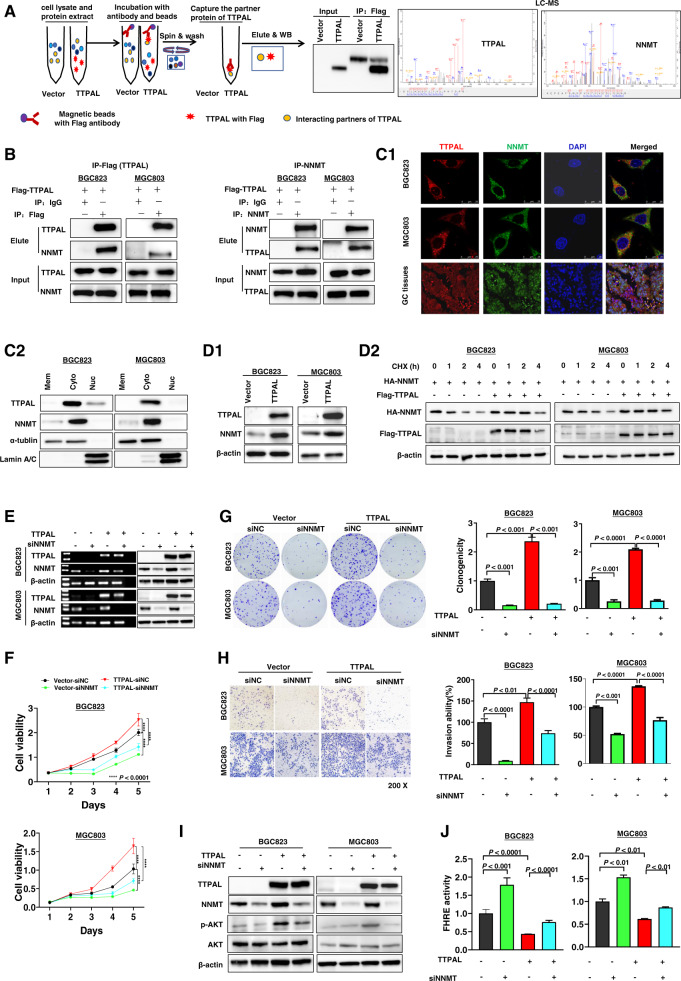
The oncogenic role of TTPAL was partially dependent on NNMT. **A** Co-immunoprecipitation (Co-IP) followed by liquid chromatography-mass spectrometry (LC-MS) identified NNMT to be a TTPAL-binding protein. **B** Co-IP followed by western blot analyses confirmed the binding between TTPAL and NNMT in BGC823 and MGC803 cells. **C** TTPAL and NNMT are mainly co-localized in cytoplasm as demonstrated by confocal immunofluorescence analysis and western blot of membrane, cytoplasmic, and nuclear fractions in BGC823 and MGC803 cells. **D** Ectopic expression of TTPAL increased the protein expression of NNMT in BGC823 and MGC803 cells by western blot. TTPAL increased the stability of NNMT in BGC823 and MGC803 cells. **E** Knockdown of NNMT in BGC823 and MGC803 cells with stable TTPAL overexpression was confirmed by RT-PCR and western blot. **F** Knockdown of NNMT significantly abolished the promoting effect of TTPAL on cell growth. **G** Knockdown of NNMT significantly abolished the promoting effect of TTPAL on cell clonogenicity. **H** Knockdown of NNMT significantly decreased invasion ability of gastric cancer cells that was promoted by TTPAL. **I** Protein expression of factors in PI3K/AKT signaling pathway in TTPAL stable expressing BGC823 and MGC803 cells with transient knockdown of NNMT by siNNMT for 48 h. **J** NNMT knockdown partially abolished the TTPAL-mediated activation of PI3K/AKT signaling as evidenced by dual luciferase reporter assay.

To further determine the downstream target of TTPAL, we performed Co-IP followed by liquid chromatograph-mass spectrometry (LC-MS). TTPAL-binding candidates were then identified by comparing the anti-TTPAL-Flag IP products of TTPAL (Flag)-overexpressed cells with those of control cells, Top 5 identified candidate genes were UGDH, DNM2, DDX46, CUL4A, and NNMT (Fig. 5A and Supplementary Table 3). Among them, NNMT was involved in PI3K/AKT signaling pathway and was the most interest target candidate of TTPAL [9, 10].

To validate the interaction between TTPAL and NNMT, IP assays were performed in the BGC823 and MGC803 cells stably transfected with TTPAL (Flag-tagged) expression. TTPAL with Flag and NNMT could be co-precipitated by each other in both cells (Fig. 5B), indicating the directly interaction between TTPAL and NNMT. The localization of the two proteins was further confirmed by confocal microscopy TTPAL co-localized with NNMT both in the cytoplasm of GC cells (BGC823 and MGC803) and GC tissues (Fig. 5C1). Western blotting of membrane, cytoplasmic and nuclear fractions, further validated that TTPAL and NNMT was mainly localized in the cytoplasm of BGC823 and MGC803 cells (Fig. 5C2). NNMT protein expression was upregulated by ectopic expression of TTPAL in BGC823 and MGC803 cells (Fig. 5D1). However, NNMT mRNA level was not changed by overexpression of TTPAL in BGC823 and MGC803 cells (Supplementary Fig. 3). We therefore assessed whether TTPAL regulated the stability of NNMT protein. We treated TTPAL or control vector-transfected cells with the protein synthesis inhibitor cycloheximide. As shown in Fig. 5D2, NNMT was more stable in the presence of TTPAL in BGC823 and MGC803 cells.

### The oncogenic role of TTPAL is partially dependent on NNMT

To investigate the effect of NNMT on the TTPAL-mediated cell proliferation and metastasis, BGC823 and MGC803 cells stably transfected with TTPAL or control vectors were co-transfected with siRNA against NNMT (Fig. 5E). NNMT knockdown significantly abolished the promoting effect of TTPAL on cell viability (Fig. 5F), clonogenicity (Fig. 5G), and invasion (Fig. 5H) abilities in both BGC823 and MGC803 cells. We further examined if TTPAL activated PI3K/AKT signaling pathway through mediating NNMT. As expected, NNMT knockdown inhibited AKT phosphorylation (Fig. 5I) and blunted the TTPAL activated PI3K/AKT signaling as evidenced by luciferase reporter assay in BGC823 and MGC803 cells (Fig. 5J). These results collectively suggested that the oncogenic role of TTPAL and activating PI3K/AKT signaling are at least in part depending on NNMT expression in GC cells.

### TTPAL promoted tumorigenicity and metastasis by inducing NNMT and activating PI3K/AKT signaling in vivo

**Fig. 6:**
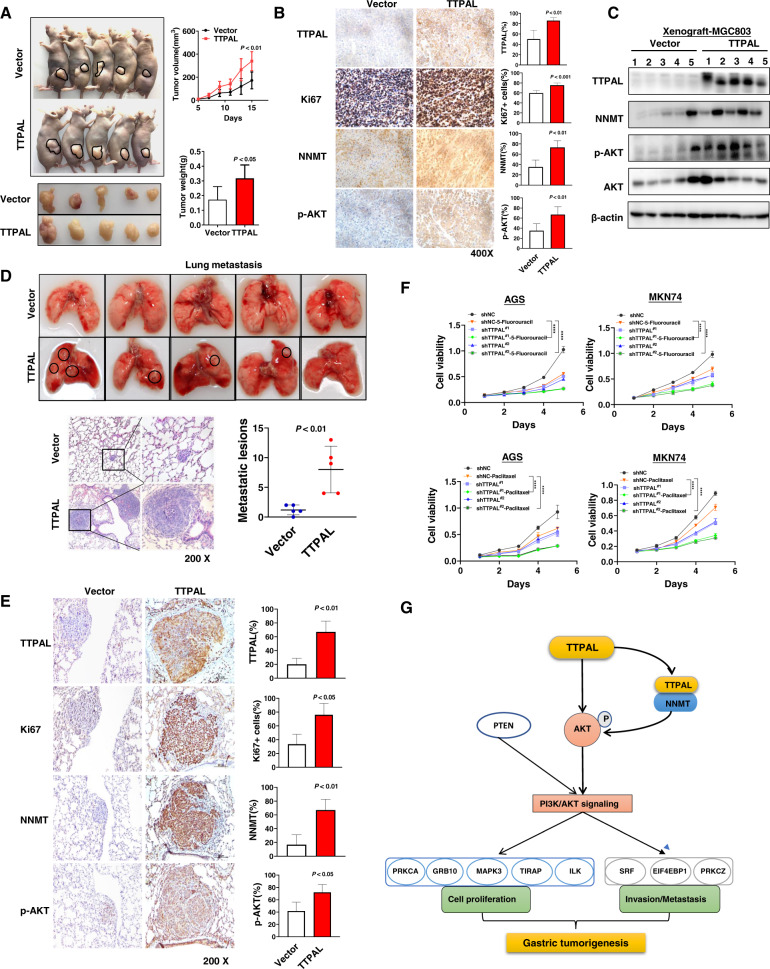
TTPAL promotes tumorigenicity and metastasis by regulating NNMT and PI3K/AKT signaling in vivo. **A** MGC803 cells stably expressing TTPAL expression promoted subcutaneous tumor growth as compared to control vector, both in terms of tumor volume over the entire assay period and tumor weight at the end point. **B** IHC staining confirmed TTPAL overexpression in MGC803 subcutaneous xenografts, which enhanced cell proliferation (by Ki-67 staining). IHC staining results also showed that TTPAL increased NNMT and p-AKT expression in xenografts. **C** Western blot analysis further confirmed that TTPAL expression in MGC803 xenografts increased the expression of NNMT and phospho-AKT. **D** Ectopic expression of TTPAL promoted experimental metastasis of MGC803 cells in vivo. Representative images of lungs and H&E staining of lung tissues from nude mice injected with TTPAL or control vector-transfected MGC803 cells. Quantitative analysis showed that TTPAL expression significantly increased the number of metastatic lesions. **E** IHC staining results also showed that TTPAL enhanced cell proliferation (by Ki-67 staining) and increased NNMT and p-AKT expression in lung metastasis. **F** Knockdown of TTPAL significantly enhanced the inhibition of cell proliferation which mediated by 5-Fluorouracil (5 μmol/l) and paclitaxel (5 nmol/l) in AGS and MKN74 cells as indicated by MTT assay. **G**. Schematic illustration of the molecular mechanism of TTPAL in PI3K/AKT signaling of GC.

To validate our in vitro findings, we subcutaneously injected MGC803 cells stably transfected with TTPAL expression vector or empty vector into the left and right dorsal flanks of nude mice, respectively. As shown in Fig. 6A, TTPAL markedly promoted the growth of tumor volume and increased tumor weight in subcutaneous xenograft models. The efficient ectopic expression of TTPAL in xenograft tumors was confirmed by immunohistochemistry (Fig. 6B). Ki-67 staining showed MGC803-TTPAL xenografts significantly promoted cell proliferation as compared to controls (Fig. 6B). Moreover, the expression of NNMT and p-AKT was dramatically increased in TTPAL-overexpressed xenografts by IHC (Fig. 6B) and western blotting (Fig. 6C), validating the molecular mechanisms identified in vitro.

We further evaluated the effect of TTPAL in GC metastasis. MGC803 cells stably transfected with TTPAL vector or empty vector were injected through tail vein of nude mice. After 4 weeks, the number of lung metastatic tumors which was confirmed histologically was significantly increased in TTPAL group as compared with control group (*p* < 0.01) (Fig. 6D), suggesting that TTPAL promotes metastasis in GC. The protein expression of Ki64, NNMT, and p-AKT was also increased in TTPAL-overexpressed metastatic tumors (Fig. 6E) validating that TTPAL promoted gastric metastasis by inducing NNMT and activating PI3K/AKT signaling.

### Knockdown of TTPAL synergized with 5-Fluorouracil and paclitaxel

With the observation that TTPAL functions as an oncogenic factor in GC, we examined if TTPAL knockdown could synergize the chemotherapeutic effects of 5-Fluorouracil and paclitaxel. As shown in Fig. 6F, knockdown of TTPAL significantly synergized 5-Fluorouracil and paclitaxel in suppressing GC cell proliferation, inferring that TTPAL might be a potential therapeutic target in GC patients.

## Discussion

In this study, we demonstrated that TTPAL was significantly upregulated in human GC at mRNA level and protein level. TTPAL is located on chromosome 20q13.12, a common region of DNA copy number gain in GC and is associated with gastric carcinogenesis [4]. TTPAL mRNA overexpression was positively correlated with its DNA copy number gain, inferring that TTPAL gene amplification contributes to its upregulation in GC. We investigated the clinical implication of TTPAL expression in GC and found that TTPAL high expression was associated with poor survival of GC patients, especially TNM stages I–III GC patients. TTPAL was an independent predictor for poor survival of GC patients (*p* < 0.05). In this connection, we investigated the function of TTPAL in GC both in vitro and in vivo. Ectopic expression of TTPAL in GC cells (BGC823 and MGC803) promoted cell proliferation and colony formation; while knockdown of TTPAL in AGS and MKN74 cells had opposite effects. TTPAL facilitated the G1-S phase transition by upregulation of the protein expression of cyclin D1 and CDK4, which are the key regulators of the transition through the G1 phase of the cell cycle. The significantly promoted cell proliferation by TTPAL was also confirmed by increased S phase cells, upregulated proliferation markers of PCNA and Ki-67 index.

In addition to growth promotion effect, ectopic expression of TTPAL significantly promoted cell migration and invasion abilities. TTPAL positively regulated EMT through upregulation of mesenchymal markers (N-cadherin and Snail) and downregulation of epithelial markers (E-cadherin). In accordance with in vitro findings, TTPAL promoted tumor growth in mouse subcutaneous xenograft models and promoted lung metastasis in tail vein injection mouse models. Moreover, we revealed that knockdown of TTPAL synergized the chemotherapeutic effects of 5-Fluorouracil and paclitaxel in GC cells. Collectively, these results indicated that TTPAL exerts oncogenic properties in GC via promoting cell proliferation and increasing metastatic abilities.

We next examined the molecular mechanism of TTPAL acting as an oncogenic factor in GC. We identified PI3K/AKT as the major downstream signaling mechanism underlying the oncogenic effect of TTPAL in GC. Ectopic expression of TTPAL activated PI3K/AKT pathway, induced phosphorylation of AKT and GSK-3β, and downregulated PTEN. TTPAL substantially enhanced the expression of PI3K/AKT target genes including SRF, EIF4EBP1, RPS6KA1, PRKCZ, ILK, TIRAP, MAPK3, GRB10, and PRKCA. These PI3K/AKT target genes are involved in proliferation [11–14], invasion, metastasis [15–17], and drug resistance of human cancers [18, 19].

Emerging evidence indicate that proteins regulate signaling pathways as part of multi-protein complexes. Here, we performed Co-IP of TTPAL followed by protein sequencing for the identification of the TTPAL interacting partner. NNMT was identified as a potential functional partner in PI3K/AKT signaling pathway [9, 10]. The direct interaction between TTPAL and NNMT was confirmed by Co-IP assays. TTPAL was co-localized in the cytoplasm with NNMT by confocal immunofluorescence assay and western blot analysis. NNMT was found upregulated following overexpression of TTPAL. These results collectively suggested that NNMT was a direct downstream interacting partner of TTPAL. Moreover, NNMT knockdown abrogated TTPAL-mediated activation of PI3K/AKT signaling and GC cell growth. Hence, TTPAL-NNMT-PI3K/AKT axis is a novel signaling cascade that cooperatively promotes GC progression (Fig. 6G).

In conclusion, we demonstrated that TTPAL was a novel oncogenic factor, which promoting GC tumorigenesis and metastasis. The oncogenic function of TTPAL was mediated by direct interaction with NNMT to activate PI3K/AKT signaling. High expression of TTPAL predicts poor prognosis for GC patients.

## Materials and methods

### Human GC samples

Three cohorts of patients with histologically confirmed GC were included in this study. Cohort I included 96 paired GC tumor tissues and adjacent non-tumor tissues for qPCR were collected from the Fourth Hospital of Hebei Medical University, Shijiazhuang, China. Cohort II included ten paired GC tumor tissues and adjacent non-tumor tissues for reverse transcription PCR were collected from Peking University Cancer Hospital, Beijing, China. Cohort III included 90 paired GC tumor tissues and adjacent non-tumor tissues for immunochemical stain was purchased commercially from the National Engineering Center For Biochip (HStmA180Su09, Outdo Biotech, Shanghai, China). Cohort IV included 375 GC tumor tissues and 28 normal controls from TCGA cohort. All our subjects provided informed consent for obtaining the study specimens. This study was approved by the Institutional Review Boards of the Fourth Hospital of Hebei Medical University and the Peking University Cancer Hospital. This study was carried out in accordance with the Declaration of Helsinki of the World Medical Association.

### GC cell lines

Six GC cell lines (AGS, BGC823, MKN45, MKN74, MGC803, HCG27) were used in this study. AGS and MKN45 were obtained from American Type Culture Collection (ATCC Manassas, VA). BGC823, HCG27, and MGC803 were obtained from Cell Research Institute, Shanghai, China. MKN74 was obtained from Japanese Collection of Research Bioresources Cell Bank, Japan. These cell lines were obtained between 2014 and 2015 and cells authentication were confirmed by short tandem repeat profiling. Cells were cultured and maintained in Dulbecco’s Modified Eagle’s medium (Gibco BRL) supplemented with 10% heat inactivated fetal bovine serum (Gibco BRL) and 1% penicillin/streptomycin according to the ATCC protocols. Cells were maintained at a 37 °C in a humidified incubator with 5% CO_2_. Routine Mycoplasma testing was performed by PCR. Cells were grown for no more than ten passages in total for any experiment.

### RNA extraction, semi-quantitative RT-PCR, and real-time PCR analyses

Total RNA was extracted from cells and tissues using TRIzol^TM^ Reagent (Thermo Fisher Scientific). cDNA was synthesized from 1 μg of total RNA using Transcriptor Reverse Transcriptase (Roche). Semi-quantitative PCR was performed by Ampli Taq Gold DNA polymerase (Applied Biosystems; Thermo Fisher Scientific). Quantitative real-time PCR was performed by SYBR Green PCR Master Mix (Applied Biosystems; Thermo Fisher Scientific) on 7500HT Fast Real-Time PCR System (Applied Biosystems; Thermo Fisher Scientific). The primers used were listed in Supplementary Table 4. Gene expression was normalized to β-actin and calculated using 2^−ΔΔCt^ method.

### TTPAL gene overexpression or knockdown

The full-length ORF of TTPAL was cloned into PLV-puro vectors (OriGene). Cell lines stably expressing TTPAL were obtained after selection with puromycin (Sigma) for at least 2 weeks. Lentivirus particles expressing TTPAL shRNA or control shRNA were produced by Gene Pharma (Shanghai, China) and then utilized to transduce cells. NNMT was knocked down using siRNA (Gene Pharma, Shanghai, China) and transfection was performed with Lipofectamine 2000 (Life Technologies). The target sequences were listed in Supplementary Table 4. Transfection efficiency was confirmed by RT-qPCR and Western blot.

### Western blotting

Proteins were separated on 10–12% SDS-polyacrylamide gel electrophoresis and transferred onto PVDF membrane. After BSA blocking, the protein-loading membrane was incubated with the primary antibody and secondary antibody. The antibodies used in this study were listed in Supplementary Table 5.

### Cell proliferation, cell cycle, wound healing, matrigel invasion assays

The cell viability, colony formation, cell cycle, cell migration, and invasion assays were performed as described previously [20]. PI3K inhibitor GDC-0941 was purchased from ApexBio (Shanghai, China) [8], 5-Fluorouracil and paclitaxel were purchased from Med Chem Express (Shanghai, China).

### In vivo subcutaneous xenograft and lung metastasis mouse models

MGC803 cells (5 × 10^6^ cells in 0.1 ml phosphate-buffered saline) stably transfected with TTPAL expression vector or empty vector were injected subcutaneously into the right and left dorsal flanks of 4 to 6-week-old male Balb/c nude mice (*n* = 5 per group). Tumor volumes were measured every 2 days using a caliper and calculated using the formula, *W*^2^ × *L*/2 (*L* = the longest diameters and *W* = the shortest diameters of the tumor). The mice were sacrificed after 2 weeks and the tumor size and tumor weight were measured. The excised tissues were either fixed in 10% neutral-buffered formalin which used for histological examination or snap frozen for molecular analyses.

For lung metastasis model, MGC803 cells stably transfected with TTPAL expression vector or empty vector (5 × 10^6^ cells in 0.1 ml PBS) were injected intravenously via the tail vein (*n* = 5). After 4 weeks, mice were sacrificed and their lungs were harvested. The lungs were sectioned and stained with HE and IHC. The number of lung metastases were counted. All experimental procedures were approved by the Animal Ethics Committee of the Chinese University of Hong Kong.

### PCR array

Human PI3K/AKT signaling pathway (Qiagen) PCR array were performed according to manufacturer’s instructions. Data analysis was performed using the RT^2^ Profiler PCR Array Data Analysis Version 3.5 software (http://pcrdataanalysis.sabiosciences.com).

### Co-immunoprecipitation (Co-IP) and liquid chromatography-mass spectrometry

Co-immunoprecipitation (Co-IP) assays were carried out as previously described [5]. Briefly, total protein from MGC803 cells stably transfected with TTPAL (Flag-tagged) expression vector or empty vector was extracted in radioimmunoprecipitation assay (RIPA) buffer supplemented with proteinase inhibitor (Novagen, Darmstadt, Germany). Immunoprecipitation was performed using anti-Flag M2 antibody (A2220, Sigma Aldrich, St. Louis, MO). The immune complexes were precipitated by Pure Proteome™ Protein A/G Mix magnetic beads (LSKMAGAG02, Millipore, Burlington, MA) overnight at 4 °C. Beads with extracted proteins were washed three times by 50 mm ammonium bicarbonate buffer and subjected to digestion by trypsin at 37 °C for 2 h (Promega, Madison, WI). Tryptic peptides were then extracted for LC-MS analysis.

### Co-immunoprecipitation of TTPAL and NNMT in GC cells

The total protein of BGC823 and MGC803 cells stably transfected with TTPAL (Flag-tagged) expression vector was extracted in RIPA buffer. Lysate (100 μg protein) and Co-IP precipitant by anti-Flag-tag, anti-NNMT antibody, or IgG were immunoblotted with either anti-NNMT or anti-TTPAL antibody to confirm the interaction of TTPAL and NNMT. The lysate (1% input, 10 μg protein) was also used as a control.

### RNA-sequencing

The total RNA from MGC803 cells stably transfected with TTPAL expression vector or empty vector was isolated using TRIzol^TM^ Reagent (Thermo Fisher Scientific). The total amount of 3 μg RNA per sample was used as input material for the RNA sample preparations. All samples had RIN values above 6.8. Sequencing libraries were generated using Illumina TruSeqTM RNA Sample Preparation Kit (Illumina, San Diego, CA). The libraries were sequenced on an Illumina HiSeq X-ten platform (Berry Genomics, Beijing, China).

### Dual-luciferase reporter assay

Briefly, cells were seeded into a 24-well plate and co-transfected with FOXO reporter and Renilla (internal control) reporter. Two days after transfection, cells were harvested, and the Firefly and Renilla luminescence were measured by the dual-luciferase reporter assay system (Promega). Reporter activity was determined as the ratio of Firefly to Renilla luciferase activity.

### Immunohistochemistry and immunofluorescence

Immunohistochemistry staining were conducted according to the procedure mentioned previously [21]. Briefly, anti-TTPAL, anti-NNMT, Anti-Phospho-AKT, and anti-Ki-67 were incubated overnight at 4 °C. The positive percentage was scored as follows: 0, no positive staining; 1, in between 1 and 25% cells; 2, in between 26 and 50% cells; 3, in between 51 and 75% cells; 4, in more than 75% cells. The staining intensity was scored as follows: 0, negative; 1, weak; 2, moderate; and 3, high intensity [21]. The final staining score was calculated as staining intensity score × percentage of positive cells. The results were evaluated blindly by two independent observers. For immunofluorescence staining, secondary fluorescent antibodies were applied for 1 h at 37 °C and sections counterstained with DAPI.

### Statistical analysis

Statistical analyses were performed using GraphPad Prism software 7.0 (GraphPad Software, CA, USA) and SPSS software (Version 22.0, IL, USA). Paired *t*-test was used to compare mRNA and protein expression of TTPAL between tumor tissues and adjacent normal tissues. Independent samples *t*-test was utilized to analyze the difference between two groups. One-way analysis of variance was used to compare means of three or more experimental groups. Crude RRs of death associated with TTPAL expression were estimated by univariate Cox proportional hazards regression model first. Multivariate Cox model was then constructed to estimate the adjusted RR for TTPAL expression. Overall survival in relation to expression was evaluated by the Kaplan–Meier survival curve and the log rank test. We analyzed the TCGA stomach adenocarcinoma dataset using the UCSC Xena tool (https://xena.ucsc.edu) for gene expression and Kaplan–Meier plotter (http://kmplot.com) for overall survival. Data were expressed as mean ± SD. *p* values < 0.05 were taken as statistical significance.

## Supplementary information

supplementary figures

Supplementary tables.
