# Correction: TTPAL promotes gastric tumorigenesis by directly targeting NNMT to activate PI3K/AKT signaling

**DOI:** 10.1038/s41388-025-03419-8

**Published:** 2025-04-24

**Authors:** Wenxiu Liu, Hongyan Gou, Xiaohong Wang, Xiaoming Li, Xiaoxu Hu, Hao Su, Shengmian Li, Jun Yu

**Affiliations:** 1 Shenzhen Research Institute, The Chinese University of Hong Kong, Shenzhen, China; 2 Department of Gastroenterology and Hepatology, The Fourth Hospital of Hebei Medical University, Shijiazhuang, China; 3 Institute of Digestive Disease and Department of Medicine and Therapeutics, State Key laboratory of Digestive Disease, Li Ka Shing Institute of Health Sciences, The Chinese University of Hong Kong, Hong Kong, China

Correction to: *Oncogene* 10.1038/s41388-021-01838-x, published online 12 October 2021

Following the publication of this article the authors noted the image of Figure 3D (MKN74-shTTPAL#2) was misplaced. The corrected version of Figure 3D (MKN74-shTTPAL#2) is shown below. The authors confirm this amendment does not affect the results or conclusions of this study and apologize for any inconvenience this may have caused.

Former Fig3:
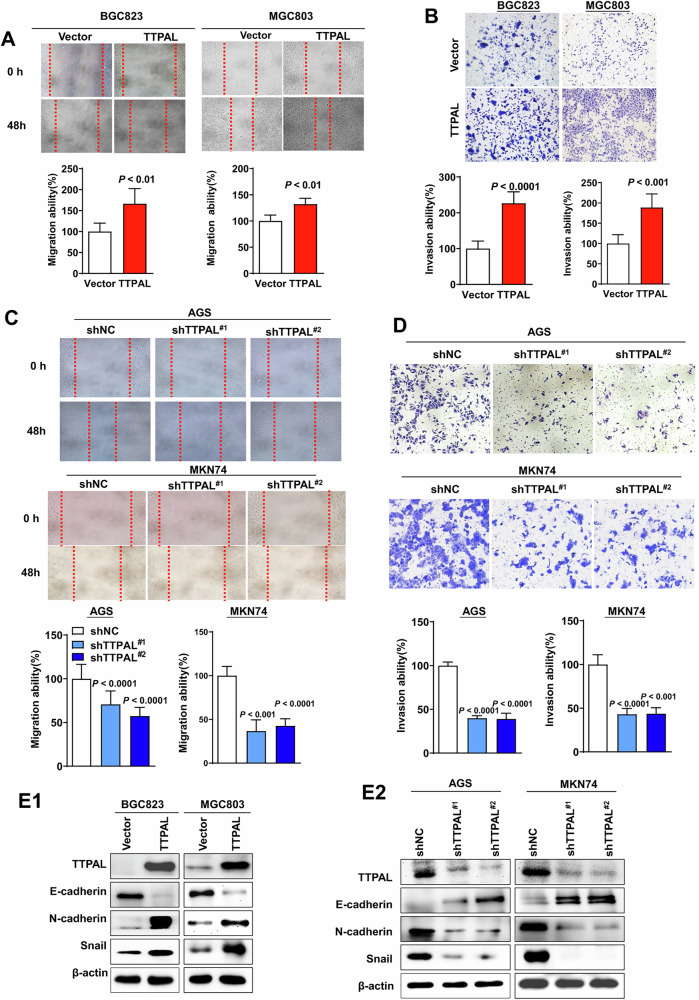


Corrected Fig3:
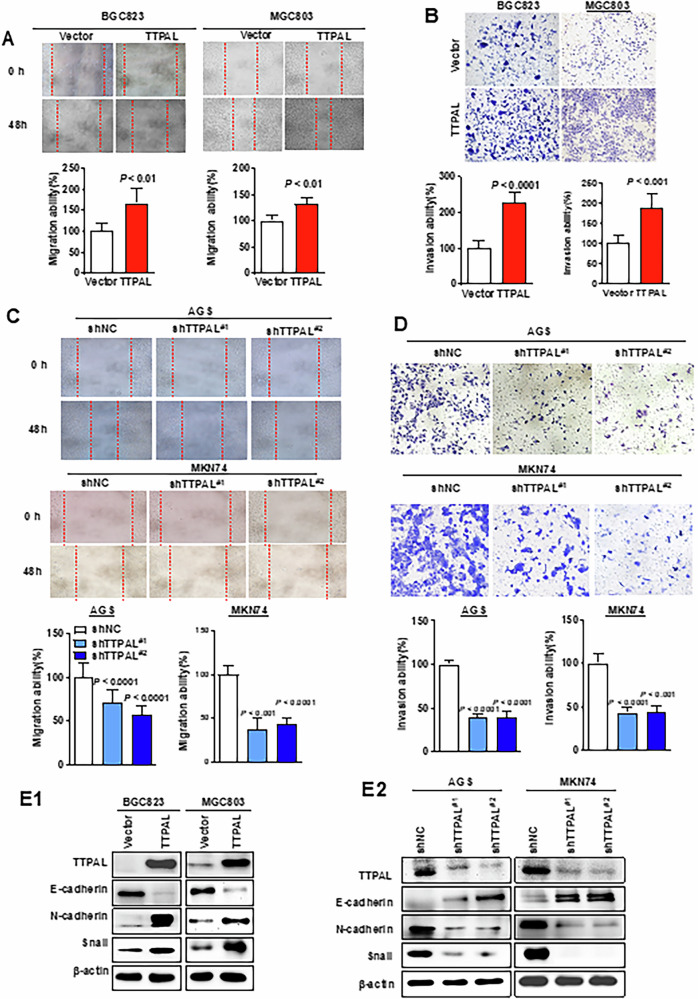


The original article has been corrected.

