# Supplementary material for: TTPAL promotes gastric tumorigenesis by directly targeting NNMT to activate PI3K/AKT signaling
Source: Oncogene. 2021 Oct 12;40(49):6666–79. doi: 10.1038/s41388-021-01838-x (PMC8660633; doi:10.1038/s41388-021-01838-x)
Supplement: Supplementary file 1 — supplementary figures [file 41388_2021_1838_MOESM1_ESM.ppt]

## Slide 1
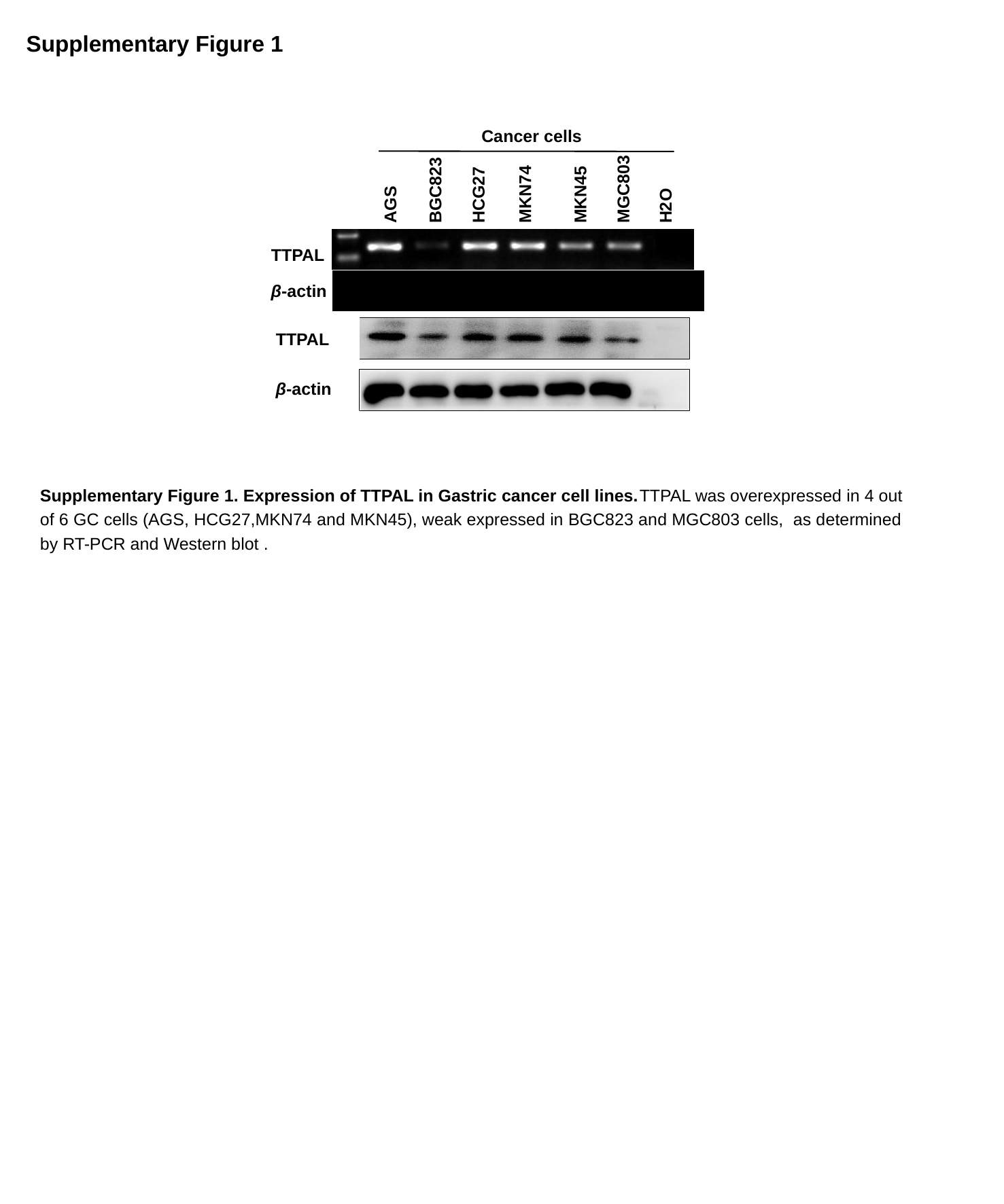

Supplementary Figure 1
Cancer cells
MGC803
BGC823
MKN74
MKN45
AGS
HCG27
H2O
TTPAL
β-actin
TTPAL
β-actin
Supplementary Figure 1. Expression of TTPAL in Gastric cancer cell lines.TTPAL was overexpressed in 4 out of 6 GC cells (AGS, HCG27,MKN74 and MKN45), weak expressed in BGC823 and MGC803 cells, as determined by RT-PCR and Western blot .

## Slide 2
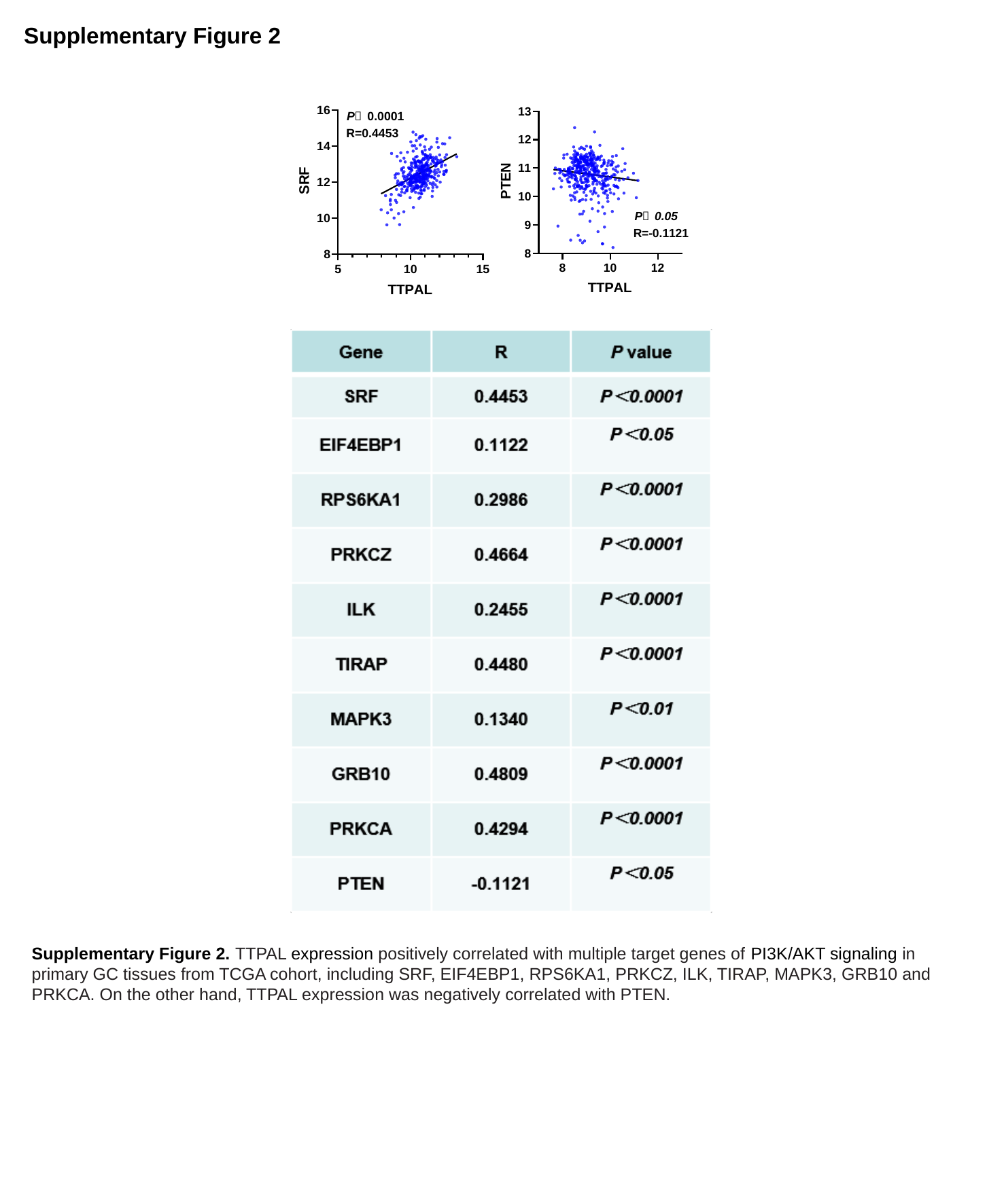

Supplementary Figure 2
Supplementary Figure 2. TTPAL expression positively correlated with multiple target genes of PI3K/AKT signaling in primary GC tissues from TCGA cohort, including SRF, EIF4EBP1, RPS6KA1, PRKCZ, ILK, TIRAP, MAPK3, GRB10 and PRKCA. On the other hand, TTPAL expression was negatively correlated with PTEN.

## Slide 3
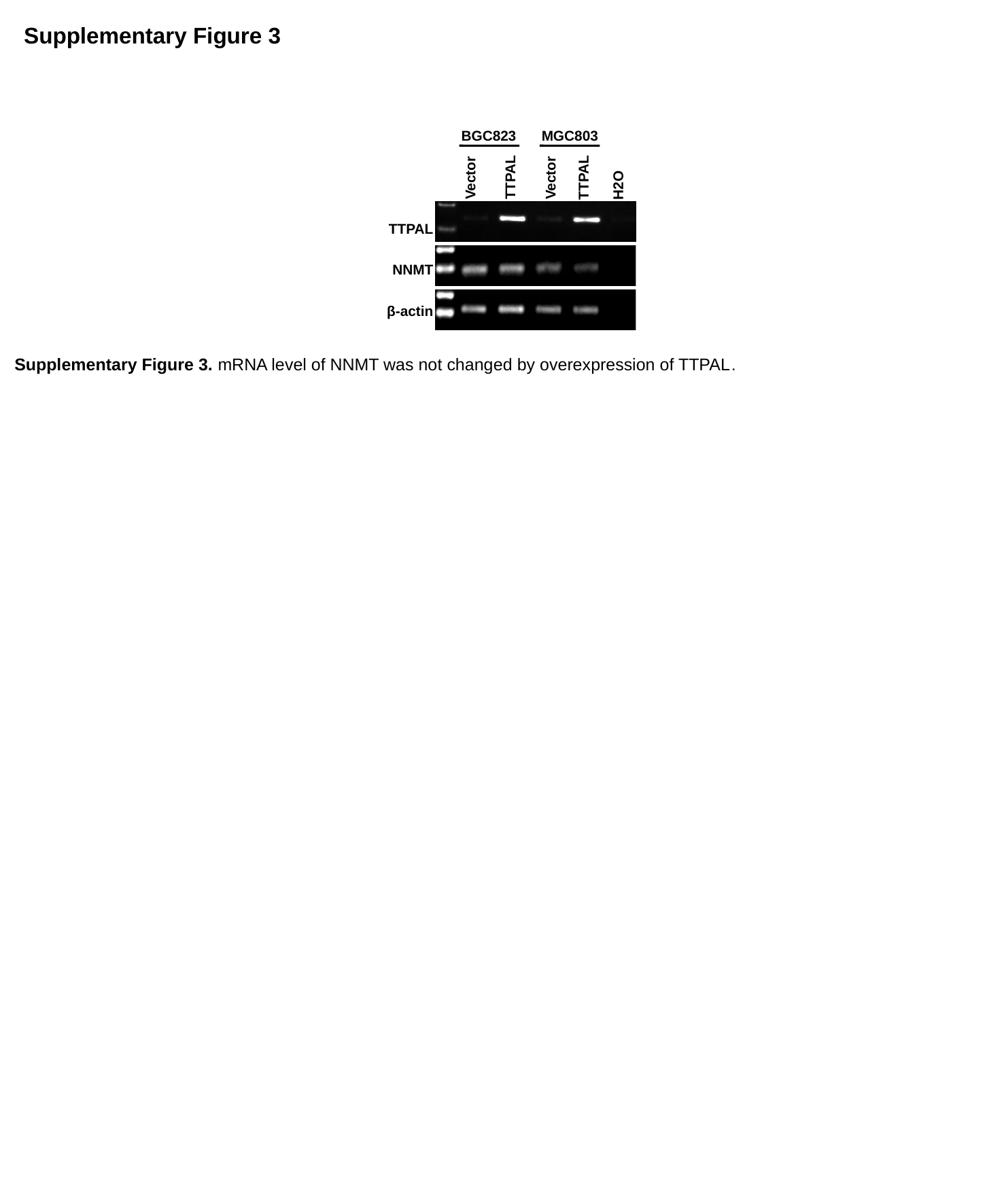

Supplementary Figure 3
BGC823
MGC803
TTPAL
H2O
TTPAL
Vector
Vector
TTPAL
NNMT
β-actin
Supplementary Figure 3. mRNA level of NNMT was not changed by overexpression of TTPAL.
