# Supplementary material for: TTPAL promotes gastric tumorigenesis by directly targeting NNMT to activate PI3K/AKT signaling
Source: Oncogene. 2021 Oct 12;40(49):6666–79. doi: 10.1038/s41388-021-01838-x (PMC8660633; doi:10.1038/s41388-021-01838-x)
Supplement: Supplementary file 2 — Supplementary tables. [file 41388_2021_1838_MOESM2_ESM.doc]

**Supplementary Table 1.** Univariate and multivariate Cox regression analyses of potential poor prognostic factors in gastric cancer patients

| **Variable** | **Univariate** | | **Multivariate** | |
| --- | --- | --- | --- | --- |
|  | RR (95% CI) | P value | RR (95% CI) | P value |
| AGE | 0.762 (0.425 to 1.366) | 0.361 |  |  |
| GENDER |  | 0.542 |  |  |
| Male | 0.911 (0.675 to 1.229) |  |  |  |
| Female | 1 |  |  |  |
| Grade |  | 0.062 |  | 0.425 |
| High | 1.614 (0.975 to 2.672) |  | 1.219 (0.719 to 2.068) |  |
| Low-moderate | 1 |  | 1 |  |
| Size |  | 0.004 |  | 0.081 |
| ≥5cm | 2.309 (1.309 to 4.074) |  | 1.687 (0.938 to 3.035) |  |
| ＜5cm | 1 |  | 1 |  |
| TNM |  | 0 |  | 0.004 |
| III-IV | 2.787 (1.619 to 4.799) |  | 2.347 (1.313 to 4.194) |  |
| I-II | 1 |  | 1 |  |
| **TTPAL expression** |  | **0.026** |  | **0.031** |
| high | 1.832 (1.075 to 3.122) |  | 1.831 (1.056 to 3.174) |  |
| low | 1 |  | 1 |  |

**Supplementary Table 2.** Clinicopathological analysis of TTPAL expression in gastric cancer

| **Variables** | **TTPAL expression (%)** | | ***P*** |
| --- | --- | --- | --- |
|  | Low | High |  |
| Ages (years) |  |  | 0.412 |
| <65 | 14(58.3) | 10(41.7) |  |
| ≥65 | 42(67.7) | 20(32.3) |  |
| Gender |  |  | 0.492 |
| Male | 13(59.1) | 9(40.9) |  |
| Female | 43(67.2) | 21(32.8) |  |
| Grade |  |  | 0.665 |
| Low-moderate | 29(63) | 17(37) |  |
| High | 27(67.5) | 13(32.5) |  |
| Size |  |  | 0.636 |
| <5cm | 21(63.6) | 12(36.4) |  |
| ≥5cm | 35(68.6) | 16(31.4) |  |
| TNM |  |  | 0.567 |
| I-II | 26(68.4) | 12(31.6) |  |
| III-IV | 30(62.5) | 18(37.5) |  |

**Supplementary Table 3.** The list of potential target genes of TTPAL identified by LC-MS

| **Gene**  **name** | **Protein function** | **Pathway** |
| --- | --- | --- |
| UGDH | The protein converts UDP-glucose to UDP-glucuronate and is a key enzyme in the uronic acid pathway. | EGFR |
| DNM2 | Regulates maturation of apoptotic cell corpse-containing phagosomes by recruiting PIK3C3 to the phagosome membrane | Homology directed repair |
| DDX46 | Plays an essential role in splicing, either prior to, or during splicing A complex formation | PI3K-Akt |
| CUL4A | Core component of multiple cullin-RING-based E3 ubiquitin-protein ligase complexes which mediate the ubiquitination of target proteins | NF-κB |
| **NNMT** | An enzyme linked to the reorganization of the methylome plays a crucial role by modulating protein methylation leading to inactivation of tumor suppressors and activation of oncogenes | PI3K-Akt |

**Supplementary Table 4. Primers, shRNA and siRNA**

| TTPAL primers | Forward 5′-AGCAGTCCATGTGGTGAATG-3′;  Reverse 5′-ATACTCCTTGGGGAGGATGC-3′. |
| --- | --- |
| NNMT primers | Forward 5’ -TGTGTGATCTTGAAGGGAACAG-3’;  Reverse 5’-CTTGACCGCCTGTCTCAAC-3’. |
| shTTPAL^#1^ target sequence | 5’ -GAAAGTGACTCTCTGAGAACC-3' |
| shTTPAL^#2^ target sequence | 5’ -GGAATGGAGACTTCGAGATGT-3' |
| siNNMT target sequence | 5’-GAAAGAGGCUGGCUACACATT-3’ |

**upplementary Table 5.** Antibodies used in this study.

| **Antibodies** | **Source** | **Identifier** | **Applications** |
| --- | --- | --- | --- |
| Anti-TTPAL | Novus | Cat#NBP1-92544 | Western Blotting 1:500  Immunocytochemistry 1:50 |
| Anti-TTPAL | Novus | Cat#NBP2-56602 | Immunofluorescence:1 100 |
| Anti-NNMT | Abcam | Cat#ab119758 | Western Blotting: 1:1000  Immunocytochemistry: 1:300  Immunofluorescence: 1:100 |
| Anti-NNMT | Santa Cruz | Cat#sc-376048 | Immunoprecipitation: 2 µg per 100µg of total protein |
| Anti-Flag | Sigma-Aldrich | Cat#A2220 | Immunoprecipitation:2 µg per 100µg of total protein |
| Anti-AKT | Cell Signaling Technology | Cat#9272 | Western Blotting: 1:1000 |
| Anti-Phospho-Akt (Ser473) | Cell Signaling Technology | Cat#4060S | Western Blotting: 1:1000  Immunocytochemistry: 1:100 |
| Anti-Phospho-GSK-3β (Ser9) | Cell Signaling Technology | Cat# 9322 | Western Blotting: 1:1000 |
| Anti-GSK-3β | Cell Signaling Technology | Cat#9315 | Western Blotting: 1:1000 |
| Anti-PTEN | Cell Signaling Technology | Cat#9559 | Western Blotting: 1:1000 |
| Anti-CyclinD1 | Cell Signaling Technology | Cat#2922 | Western Blotting: 1:1000 |
| Anti-CDK4 | Cell Signaling Technology | Cat#12790 | Western Blotting: 1:1000 |
| Anti-PCNA | Cell Signaling Technology | Cat#13110 | Western Blotting: 1:1000 |
| Anti-E-cadherin | Cell Signaling Technology | Cat#14472 | Western Blotting: 1:1000 |
| Anti-N-cadherin | Cell Signaling Technology | Cat#13116 | Western Blotting: 1:1000 |
| Anti-Snail | Cell Signaling Technology | Cat#3879 | Western Blotting: 1:1000 |
| Anti-α-tublin | Cell Signaling Technology | Cat#2125 | Western Blotting: 1:1000 |
| Anti-LaminA/C | Cell Signaling Technology | Cat#4777 | Western Blotting: 1:2000 |
| Anti-β-actin | Cell Signaling Technology | Cat#3700 | Western Blotting: 1:1000 |
